# The Burden of Cardiovascular Disease from Air Pollution in Rwanda

**DOI:** 10.5334/aogh.4322

**Published:** 2024-01-08

**Authors:** Gabriella Taghian, Samantha Fisher, Thomas C. Chiles, Agnes Binagwaho, Philip J. Landrigan

**Affiliations:** 1 Global Pollution Observatory on Planetary Health, Boston College, 140 Commonwealth Avenue, Chestnut Hill, MA 02467, US; 2 Wake Forest School of Medicine, Winston-Salem, NC, US; 3 School of Public Health, New York University, New York, NY, US; 4 Department of Biology, Boston College, 140 Commonwealth Avenue, Chestnut Hill, MA 02467, US; 5 University of Global Health Equity, Butaro, RW; 6 Department of Biology and the Global Pollution Observatory for Planetary Health, Boston College, 140 Commonwealth Avenue, Chestnut Hill, MA 02467, US; 7 Centre Scientifique de Monaco, Monaco, MC

**Keywords:** Cardiovascular disease, air pollution, epidemiologic transition, Rwanda, Africa

## Abstract

**Background:**

Rwanda, like many countries in sub-Saharan Africa, is still relatively early in development. Industrialization and urbanization are major drivers of the county’s economic growth. Rwanda is also undergoing an epidemiological transition, from a pattern of morbidity and mortality dominated by infectious diseases to a pattern shaped by non-communicable diseases (NCDs). The rise in NCDs is due, in part, to increasing exposures to environmental hazards. These include emissions from the growing number of motor vehicles and toxic occupational exposures. Cardiovascular disease (CVD) is now an increasingly important cause of death in Rwanda, and ambient air pollution is a CVD risk factor of growing importance.

**Objectives:**

To quantify the burden of CVD attributable to air pollution in Rwanda and identify opportunities for prevention and control of air pollution and pollution-related disease.

**Methods:**

We relied on the 2019 Global Burden of Disease (GBD) study for information on levels, sources, and trends in household and ambient air pollution and the burden of pollution-related disease in Rwanda. Information on pollution sources was obtained from the Health Effects Institute State of Global Air 2019 report.

**Findings:**

An estimated 3,477 deaths (95% Uncertainty Interval [UI]: 2,500–4,600) in Rwanda in 2019 were attributable to air pollution-related CVD. Of these, 689 (UI: 283–1,300) deaths were from ambient air pollution-related CVD, while 2,788 (UI: 1,800–3,800) deaths were from household air pollution-related CVD.

**Conclusion:**

Rwanda is experiencing increased rates of disease and premature death from NCDs, including CVD, as the country grows economically. While household air pollution is still the top pollution-related cause of disease and premature death, rising levels of ambient air pollution are an increasingly important CVD risk factor.

**Recommendation:**

Actions taken now to curb rising levels of ambient air pollution will improve health, reduce CVD, increase longevity, and produce great economic benefit for Rwanda. The single most effective intervention against air pollution will be a rapid nationwide transition to renewable energy. We recommend additionally that Rwanda prioritize air pollution prevention and control, establish a robust, nationwide air monitoring network, support research on the health effects of air pollutants, and build national research capacity. The allocation of increased resources for rural and urban public health and health care will complement air pollution control measures and further reduce CVD. To incentivize a rapid transition to renewable energy in Rwanda and other nations, we recommend the creation of a new Global Green Development Fund.

## Introduction

**Figure 1:**
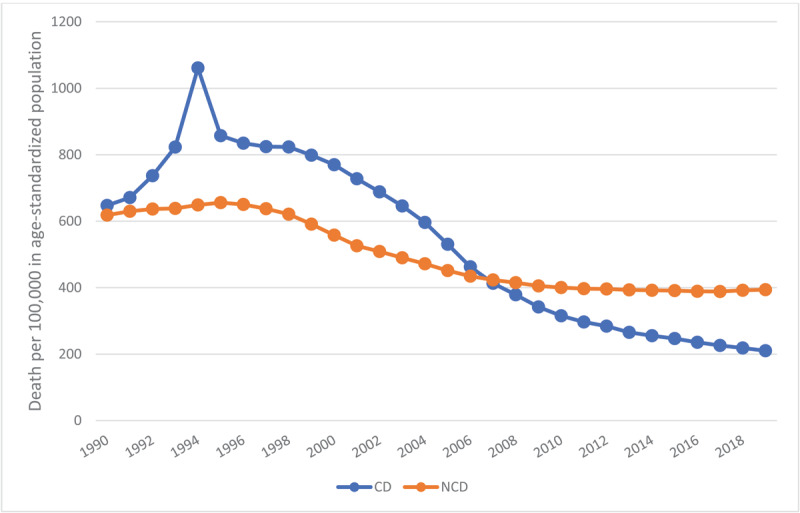
Age-Standardized Death Rates from Communicable and Non-Communicable Diseases, Rwanda, 1990–2019. *Source:* IHME.

Rwanda is a small, densely populated, landlocked, mountainous country in equatorial Africa with a population of 14 million people and a gross domestic product (GDP) of $10.35B [1,2,3]. Despite its classification by the World Bank as a low-income country, Rwanda has registered an average GDP growth rate of approximately 8.0 percent per year over the last two decades [2]. Industrialization and urbanization are major contributors to this economic growth, and the country’s industrial production growth rate in 2021 was estimated to be 13.4%, ranking it 19^th^ in the world [3]. Concomitant with this economic growth, the country is undergoing an epidemiological transition, from a pattern of disease and mortality dominated by infectious diseases to a pattern shaped by chronic non-communicable diseases (NCDs) (Figure 1).

Air pollution is a major risk factor for mortality and morbidity worldwide [4,5,6]. The Global Burden of Disease (GBD) study estimated that air pollution was responsible for 6.7 million deaths in 2019 [5]. Approximately 4.1 million of these deaths were the result of ambient air pollution, and 2.3 million were due to household air pollution. In Africa, air pollution was responsible for 1.1 million deaths in 2019. Reflecting the relatively early economic development of most sub-Saharan African countries, household air pollution was the predominant source of pollution-related mortality and accounted for 697,000 of these deaths, while ambient air pollution accounted for 394,000 [7]. Deaths due to household air pollution are declining as countries develop, while deaths attributable to ambient air pollution are on the rise [5].

Ambient air pollution has been associated with multiple NCDs, including cardiovascular disease (CVD), diabetes, lung cancer, chronic obstructive pulmonary disease (COPD), and dementia among adults [5,8,9,10,11,12,13,14]. The CVD mortality rate is 2.5 times higher in low- and middle-income countries than in high-income countries [11]. The Rwandan Ministry of Health (2020) reports that NCDs, including CVD, are the second leading cause of death in Rwanda [15], accounting for 34.7% of deaths [11].

Rwanda is at a critical inflection point in its economic development. Within the past decade, Rwanda, like many African countries, has seen a deterioration in ambient air quality, reflected in a steady rise in PM_2.5_ levels [5,7,16]. In 2017, the Health Effects Institute [4] and the Institute for Health Metrics and Evaluation reported that the annual PM_2.5_ mean exposure in Rwanda in 2019 was 43.21 μg/m^3^ [16]. This is substantially higher than the WHO air quality guideline of 5 μg/m^3^. Principal sources of ambient air pollution in urban areas of Rwanda include emissions from motor vehicles and industries, whereas in residential and rural areas, the major sources are the burning of biomass fuels from wood- and charcoal-based cookstoves and the open burning of crop residues [4,7,17]. Rwanda is a mountainous country, and topographical and meteorological conditions are key contributors to the spatial distribution of air pollution [18].

Air pollution is the second leading risk factor for premature death in Rwanda, after malnutrition, accounting for more than 8% of deaths in 2017 [16]. A recent report found that the loss in economic output due to air pollution-related morbidity and mortality was $349 million in 2019, accounting for 1.9% of the country’s GDP [7]. PM_2.5_ pollution also causes a substantial reduction in cognitive function and was responsible in 2019 for an estimated loss of 18.5 million intelligence quotient (IQ) points among Rwandan children younger than 10 years of age [7].

The government of Rwanda has taken steps to identify major air pollution sources and improve national air quality monitoring, particularly in the capital city of Kigali. Despite these efforts, ambient air pollution in Rwanda is a growing threat to human health, economic development, and human capital [7]. There is a major need to understand how changing patterns of ambient air pollution are driving the rise of NCDs in Rwanda.

The goals of this study are to assess the current burden of disease attributable to air pollution in Rwanda and to offer recommendations for air pollution control and prevention of pollution-related human disease.

## Materials and Methods

To assess the burden of disease due to air pollution in Rwanda, we relied primarily on data from the 2019 GBD study [4] coordinated by the Institute of Health Metrics and Evaluation. The GBD study is a comparative assessment of disease burden in 195 countries and territories, covering the years 1990–2019 [19]. The GBD study includes information on multiple risk factors for disease, including air pollution, and enables the estimation of the number of CVD deaths due to air pollution for each country. A detailed summary of the GBD methodology as applied to estimates of CVD morbidity and mortality is presented in the report by Roth et al [8]. Using the GBD risk factor database, we extracted information on the estimated numbers of CVD deaths in Rwanda attributable to both ambient air pollution and household air pollution in 2019.

Further information on air pollution sources in Rwanda was obtained from the Health Effects Institute’s State of Global Air/2019 report [16] and the World Health Organization’s Global Health Observatory [20,21].

## Results

### Cardiovascular Disease in Rwanda

Rwanda has a population of 14.09 million people, with an annual growth rate of approximately 2.1% (est. 2023) [1]. The population is young, with children 14 years of age and younger making up 39.9% of the total in 2019. The fertility rate is declining and fell from 5.7 to 3.99 children per woman between 2000 and 2018, while the average life expectancy at birth increased from 47 years to 69 years [1,3]. An Institute for Health Metrics and Evaluation report indicates that NCDs represent significant contributors to health loss in Rwanda, accounting for 42% of DALYs in 2019, up from 19% in 1990 [20].

**Figure 2:**
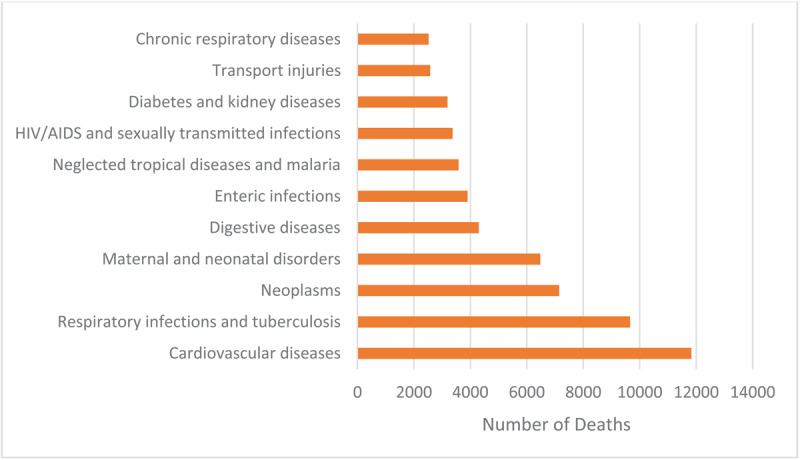
Number of Deaths by Cause, Rwanda, 2019. *Source:* IHME.

CVD was the leading cause of death in Rwanda in 2019, resulting in 11,826 deaths (Figure 2). Respiratory infections and tuberculosis accounted for 9,658 deaths and were the second leading cause of death, followed by neoplasms (7,143), maternal and neonatal disorders (6,474), digestive diseases (4,297), enteric infections (3,901), neglected tropical diseases and malaria (3,583), HIV/AIDS and sexually transmitted infections (3,370), diabetes and kidney diseases (3,189), transport injuries (2,578), and chronic respiratory diseases (2,520).

CVD deaths in Rwanda are increasing. In 1990, CVD was associated with 10,440 deaths (95% Uncertainty Interval [UI]: 8,687–12,408). This number fell to 8,146 deaths ([UI]: 6,905–9,534) in 2009 but has since rebounded and increased by 30% from 2009 to 2019. The number of disability-adjusted life years (DALYs) attributable to CVD in Rwanda in 2019 was 310,453 years ([UI]: 252,000–385,000). CVD is most highly prevalent in older members of the population (80+ years), with a death rate of 4,869 deaths per 100,000, almost double that in the next most highly affected age group, 75–79 years.

### Air Pollution in Rwanda

**Figure 3:**
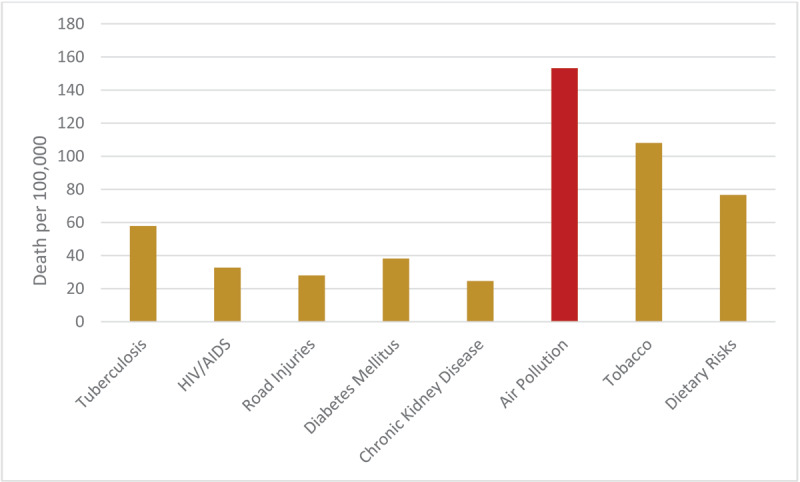
Age Standardized Death Rates by Major Causes and Risk Factors, Rwanda, 2019. *Source:* IHME.

Ambient air pollution is increasing across most of Africa as the continent urbanizes and industrializes. The estimated number of deaths from all types of air pollution in Rwanda in 2019 was 9,286 ([UI]: 7,500–11,400). This far exceeds the numbers attributable to HIV/AIDS (3,065), diabetes mellitus (1,916), chronic kidney disease (1,266), road injuries (2,401), tuberculosis (3,960), tobacco (5,450), and dietary risks (3,636) (Figure 3).

Deaths from all air pollution in Rwanda spiked in 1997, causing 12,743 deaths, followed by a rapid decrease, bottoming out in 2013 at 8,501 deaths. Since 2013, there has been a steady rise. The 9,286 air-pollution-related deaths estimated in 2019 include 1,757 deaths ([UI]: 800–3,200) from ambient fine particulate (PM_2.5_) air pollution and 7,468 deaths ([UI]: 5,300–9,900) from household air pollution. Household air pollution was responsible for 80.4% of air pollution-related deaths in Rwanda in 2019. Deaths from household air pollution are beginning to plateau, while deaths from ambient air pollution increased by 14% from 2017 to 2019.

### Cardiovascular Disease and Air Pollution

**Figure 4:**
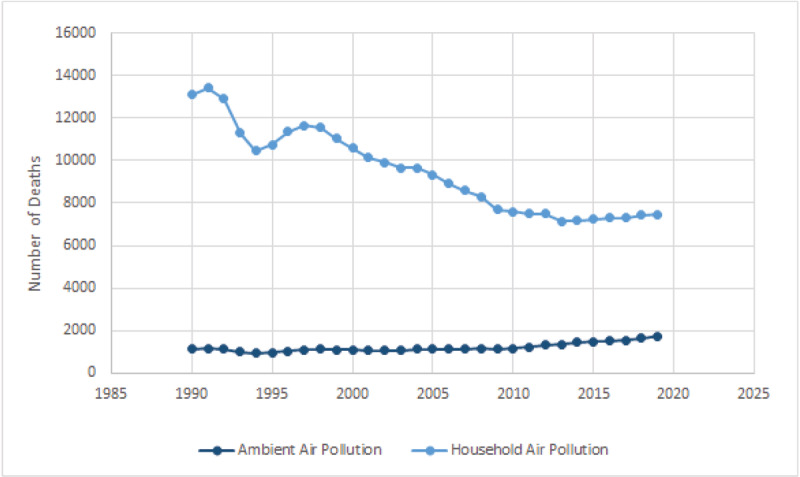
Transition in Deaths from Specific Types of Air Pollution, All Ages, Rwanda, 1990–2019. *Source:* IHME.

In 2019, approximately 3,477 deaths ([UI]: 2,500–4,600) in Rwanda were attributable to air pollution-related CVD. Of these, 689 ([UI]: 283–1,300) deaths are from ambient air pollution-related CVD, while the other 2,788 ([UI]: 1,800–3,800) deaths are from household air pollution-related CVD. Deaths from all air pollution-related CVD increased by 41% from 2008 to 2019. (Figure 4). Deaths from air pollution-related CVD are most prevalent in the 65–69 age group.

The majority of deaths from ambient air pollution-related CVD are male (56%), while men comprised only 45% of the CVD deaths from household air pollution. The highest number of deaths from household air pollution-related CVD in Rwanda in 2019 were in females 70 years of age or older, with a death number of 813. This gender imbalance likely reflects the disproportionate exposure of women to household air pollution associated with cooking on poorly ventilated indoor stoves.

## Discussion

Ambient air pollution is increasing across Africa [22] and has become a major cause of disease, disability, and premature death [4,5,7]. While household air pollution remains the predominant form of air pollution across the African continent, it is in decline, while ambient air pollution and the diseases it causes are on the rise [4]. Ambient air pollution represents a major threat to African countries’ economies, human capital, and future development prospects [7]. A recent study underscores the contribution of increased air pollution levels resulting from combustion emissions and unfavorable weather conditions to the rapid deterioration of East Africa’s air quality [18].

Increases in ambient air pollution in African countries closely parallel the national trajectories of economic development and fossil fuel combustion. To advance development in Rwanda, the Government of Rwanda launched a 20-year economic development strategy in 2000 (VISION 2020), with the goal of transforming Rwanda into a middle-income economy [23]. To achieve this goal, the country has undergone an accelerated push towards technology and industrialization. Rwanda’s air quality has deteriorated in parallel with this development and is projected to deteriorate further in the near future as the country becomes more industrialized and its population continues to grow.

There is great danger in economic development based on fossil fuels. Countries that have powered their economic growth through massive combustion of coal, oil, and gas have increased national income and improved living standards, but these gains have come at the cost of high levels of ambient air pollution and pollution-related disease. They have also resulted in substantial economic costs due to the reduced economic productivity and increased healthcare expenditures of populations burdened with high rates of pollution-related disease. These costs pull down national economies and slow economic development. By impairing children’s cognitive function and reducing their IQ, these exposures reduce human capital and undermine the trajectories of national growth [5,24,25,26].

The Government of Rwanda has acted to improve air quality countrywide and reduce pollution-related diseases, most notably through the passage of the 2016 Air Quality Law. This law sets standards for the regulation and prevention of air pollution in Rwanda [27]. The development and enforcement of air pollution control laws such as the 2016 Air Quality Law is a proven effective strategy for reducing pollution and preventing pollution-related disease and death [29]. In the United States, passage of the Clean Air Act in 1970, which authorized the development of comprehensive federal and state regulations to limit emissions from both stationary sources and mobile sources, has reduced air pollution levels by 77% and brought about a reduction in rates of air pollution-related morbidity and death [30].

Air pollution control laws are also highly cost-effective, and they promote economic growth in countries at all income levels by improving health, increasing longevity, and enhancing children’s IQ, thus enabling advances in economic productivity, national security, and human well-being [5,7]. It is estimated that every dollar invested in air pollution control in the United States since 1970 has yielded an economic return of $30 (USD) [30].

Further measures to control air pollution that could be taken in Rwanda to complement the 2016 Air Quality Law could include education programs that raise public awareness in rural and urban areas of air pollution hazards to human health and the environment, the adoption of standards for cleaner vehicles, and routine inspections of older motor vehicles.

To further address the rising rates of NCDs, Rwanda has recently adopted a national NCD prevention and control strategy. This National Strategy and Costed Action Plan for the Prevention and Control of Non-Communicable Diseases spans a five-year period from July 2020 to June 2025 [15]. The actions taken under this strategy will be powerful complements to air pollution control in reducing CVD risk.

Beyond these actions already taken, Rwanda has a unique opportunity to leapfrog over the negative consequences of fossil-fuel-based economic development through a preemptive, wide-scale national transition to clean energy. The transition to renewable energy through investment in hydropower plants, wind farms, solar power systems, and the electric grid is already beginning in the country and has the potential to be a key intervention against both climate change and air pollution.

A major challenge to rapid energy transition is the high up-front cost. A mechanism to overcome this challenge and incentivize a preemptive transition to renewable energy in Rwanda and other countries could be the creation of a new Global Green Development Fund under the auspices of the United Nations or a UN agency. Investments in clean energy made with the support of such a fund would improve air quality, slow global warming, and pay for themselves many times over in subsequent decades through reducing healthcare costs and increasing economic productivity.

We offer the following recommendations for controlling air pollution (both ambient and household) and for mitigating the burden of CVD and other NCDs associated with air pollution in Rwanda:

1. **Invest in clean energy and minimize dependence on fossil fuels**. This is the single most effective strategy for reducing air pollution, pollution-related disease, and pollution-related economic losses. Such investment will create well-paying jobs in wind, solar, and geothermal energy, and these jobs will remain in the country. It represents a long-term investment in Rwanda’s future.
2. **Prioritize air pollution prevention and control**. Air pollution can be reduced through a judicious combination of incentives and penalties that encourage both the public and private sectors to reduce pollutant emissions and transition to clean, renewable energy sources [9].
3. **Establish a robust, nationwide air monitoring network**. Air monitoring and identification of pollution sources are two essential prerequisites to pollution control. Establishment of a national network of low-cost real-time, multi-pollutant monitors, as has recently been piloted in a long-term, ground-based monitoring study in Kigali, may represent a way forward [31].
4. **Support research on the health effects of air pollutants and build national research capacity**. The University of Rwanda and the University of Global Health Equity are well positioned to become leaders in research on the health effects of household and ambient air pollution in Africa and to train a new generation of environmental health researchers who will safeguard health and save lives in Rwanda and across the continent.
5. **Allocation of increased resources for rural and urban public health and health care**. Proactive, community-based educational interventions have proven highly effective in encouraging the adoption of healthy behaviors and reducing CVD risk [28,32,33,34]. An expansion in the number of community-based health workers in Rwanda will support community-based health education across the country [28].
6. **Create Global Green Development Fund** under the auspices of the United Nations or a UN agency to incentivize a rapid, wide-scale transition to renewable energy.

## Data Accessibility Statement

Data for CVD and air pollution-related disease and death was gathered from the 2019 GBD database using the GBD Results Tool. This can be found at http://ghdx.healthdata.org/gbd-results-tool.
